# Supplementary material for: Label-free identification of protein aggregates using deep learning
Source: Nat Commun. 2023 Nov 28;14:7816. doi: 10.1038/s41467-023-43440-7 (PMC10684545; doi:10.1038/s41467-023-43440-7)
Supplement: Supplementary file 3 — Reporting Summary [file 41467_2023_43440_MOESM3_ESM.pdf]

nature portfolio

Corresponding author(s): Prof. Kristin Grußmayer, Prof. Hilal Lashuel, Prof. Aleksandra Radenovic

Last updated by author(s): Oct 23, 2023

Reporting Summary

Nature Portfolio wishes to improve the reproducibility of the work that we publish. This form provides structure for consistency and transparency in reporting. For further information on Nature Portfolio policies, see our Editorial Policies and the Editorial Policy Checklist.

Statistics

For all statistical analyses, confirm that the following items are present in the figure legend, table legend, main text, or Methods section.

n/a	Confirmed	
☐	☒	The exact sample size (*n*) for each experimental group/condition, given as a discrete number and unit of measurement
☐	☒	A statement on whether measurements were taken from distinct samples or whether the same sample was measured repeatedly
☐	☒	The statistical test(s) used AND whether they are one- or two-sided<br>*Only common tests should be described solely by name; describe more complex techniques in the Methods section.*
☒	☐	A description of all covariates tested
☒	☐	A description of any assumptions or corrections, such as tests of normality and adjustment for multiple comparisons
☐	☒	A full description of the statistical parameters including central tendency (e.g. means) or other basic estimates (e.g. regression coefficient) AND variation (e.g. standard deviation) or associated estimates of uncertainty (e.g. confidence intervals)
☐	☒	For null hypothesis testing, the test statistic (e.g. *F*, *t*, *r*) with confidence intervals, effect sizes, degrees of freedom and *P* value noted<br>*Give P values as exact values whenever suitable.*
☒	☐	For Bayesian analysis, information on the choice of priors and Markov chain Monte Carlo settings
☒	☐	For hierarchical and complex designs, identification of the appropriate level for tests and full reporting of outcomes
☐	☒	Estimates of effect sizes (e.g. Cohen's *d*, Pearson's *r*), indicating how they were calculated

Our web collection on statistics for biologists contains articles on many of the points above.

Software and code

Policy information about availability of computer code

Data collection	Custom LabVIEW code (2021 version) was used for data acquisition on our multi-modal, multi-plane microscope. The automatic scanning mode and the microscope are described in the manuscript.
Data analysis	Data analysis was performed using custom MATLAB (R2021a), available here: https://c4science.ch/source/TomPhaseRet/, to retrieve the phase information from the brightfield images and produce quantitative phase images. We used Fiji (v2.9.0) scripts to produce maximum z-projection fluorescence images, to segment these images using Otsu thresholding and produce the labels for pixel classification, to prepare the color-coded maximum z-projection phase image shown in Fig. 2a, and for the image processing in Fig. 2b. Fiji was also used to segment network predictions and produce masks which are used to measure the area, circularity or dry mass of aggregates. Python (v3.7) was used for data analysis and plotting. Python was also used for neural network training, along with Tensorflow 2.8, Keras 2.8, CUDA 11.1 and CUDNN 8.1. Code related to neural network training and usage is available on GitHub (http://github.com/kibb/LINA).

For manuscripts utilizing custom algorithms or software that are central to the research but not yet described in published literature, software must be made available to editors and reviewers. We strongly encourage code deposition in a community repository (e.g. GitHub). See the Nature Portfolio guidelines for submitting code & software for further information.

Data

Policy information about availability of data

All manuscripts must include a data availability statement. This statement should provide the following information, where applicable:
- Accession codes, unique identifiers, or web links for publicly available datasets
- A description of any restrictions on data availability
- For clinical datasets or third party data, please ensure that the statement adheres to our policy

All data needed to evaluate the conclusions in the paper are present in the manuscript and/or the supplementary information. Additional data related to this paper are available from the corresponding authors upon request.

Research involving human participants, their data, or biological material

Policy information about studies with human participants or human data. See also policy information about sex, gender (identity/presentation), and sexual orientation and race, ethnicity and racism.

Reporting on sex and gender	N/A
Reporting on race, ethnicity, or other socially relevant groupings	N/A
Population characteristics	N/A
Recruitment	N/A
Ethics oversight	N/A

Note that full information on the approval of the study protocol must also be provided in the manuscript.

Field-specific reporting

Please select the one below that is the best fit for your research. If you are not sure, read the appropriate sections before making your selection.

[x] Life sciences [ ] Behavioural & social sciences [ ] Ecological, evolutionary & environmental sciences

For a reference copy of the document with all sections, see nature.com/documents/nr-reporting-summary-flat.pdf

Life sciences study design

All studies must disclose on these points even when the disclosure is negative.

Sample size	Sample sizes were determined based on previous studies using similar methodologies and were chosen to be large enough to avoid overfitting (alongside other methods for avoiding it, such as using EarlyStopping). For example: https://www.nature.com/articles/s41592-018-0111-2#Sec2; this study uses fewer than 100 examples for training. We used > 900 for training and validation, to make sure we have the best performance we can have, and also quantified the reliance of the performance on the amount of training data. The particular number of examples needed for deep learning model training depends on the particular use case and is difficult to precisely determine a priori.
Data exclusions	No data were excluded from the analysis.
Replication	Independent experiments were performed to verify the findings (minimum 3 independent experiments) with all attempts at replication successful.
Randomization	No experiments requiring randomization were conducted. There was no human or animal testing done.
Blinding	Blinding was not relevant to our study as we did not perform assays that depend on the experimenter.

Reporting for specific materials, systems and methods

We require information from authors about some types of materials, experimental systems and methods used in many studies. Here, indicate whether each material, system or method listed is relevant to your study. If you are not sure if a list item applies to your research, read the appropriate section before selecting a response.

Materials & experimental systems

n/a	Involved in the study
	☒ Antibodies
	☒ Eukaryotic cell lines
☒	Palaeontology and archaeology
☒	Animals and other organisms
☒	Clinical data
☒	Dual use research of concern
☒	Plants

Methods

n/a	Involved in the study
☒	ChIP-seq
☒	Flow cytometry
☒	MRI-based neuroimaging

Antibodies

Antibodies used

Primary antibody: Anti-Huntingtin raised against the Proline-rich domain (PRD) (MAB5492, Millipore)
Secondary antibody: donkey anti-mouse Alexa488 (Life Technologies, Switzerland)

Validation

The antibodies are from commercial sources and have been validated by the vendors. Their validation data are available on the manufacturers' website:
MAB5492 Huntingtin antibody: https://www.merckmillipore.com/CH/de/product/Anti-Huntingtin-Antibody-a.a.-1-82,MM_NF-MAB5492

Eukaryotic cell lines

Policy information about cell lines and Sex and Gender in Research

Cell line source(s)

HEK293 cells: ATCC, [HEK-293T/17] (ATCC® CRL-11268).
HeLa cells: ATCC, (ATCC® CCL-2).

Authentication

HEK293 cells were identified by the vendor: https://www.atcc.org/products/crl-11268 (karyotyping) and were not authenticated for this study in particular.

Mycoplasma contamination

The HEK293 cells were tested negative to mycoplasma contamination. HeLa cells were not tested for mycoplasma after purchase from the vendor.

Commonly misidentified lines
(See ICLAC register)

No commonly misidentified cell lines were used in the study.
